# RNAi Suppression of *LEAFY* Gives Stable Floral Sterility, and Reduced Growth Rate and Leaf Size, in Field-Grown Poplars

**DOI:** 10.3390/plants10081594

**Published:** 2021-08-03

**Authors:** Amy L. Klocko, Amanda L. Goddard, Jeremy R. Jacobson, Anna C. Magnuson, Steven H. Strauss

**Affiliations:** 1 Department of Biology, University of Colorado Colorado Springs, Colorado Springs, CO 80918, USA; aklocko2@uccs.edu; 2 Department of Forest Ecosystems and Society, Oregon State University, Corvallis, OR 97331, USA; Amanda.Goddard@oregonstate.edu (A.L.G.); jeremy.jacobson3402@gmail.com (J.R.J.); anna.magnuson@oregonstate.edu (A.C.M.)

**Keywords:** floral development, RNAi, *LEAFY*, Populus, sterility, containment

## Abstract

The central floral development gene *LEAFY* (*LFY)*, whose mutation leads to striking changes in flowering and often sterility, is commonly expressed in non-floral structures; however, its role in vegetative development is poorly understood. Sterility associated with suppression of *LFY* expression is an attractive means for mitigating gene flow by both seeds and pollen in vegetatively propagated forest trees, but the consequences of its suppression for tree form and wood production are unclear. To study the vegetative effects of RNAi suppression of *LFY*, we created a randomized, multiple-year field study with 30–40 trees (ramets) in each of two sterile gene insertion events, three transgenic control events, and a wild-type control population. We found that floral knock-down phenotypes were stable across years and propagation cycles, but that several leaf morphology and productivity traits were statistically and often substantially different in sterile vs. normal flowering RNAi-*LFY* trees. Though trees with suppressed *LEAFY* expression looked visibly normal, they appear to have reduced growth and altered leaf traits. *LFY* appears to have a significant role in vegetative meristem development, and evaluation of vegetative impacts from *LFY* suppression would be prudent prior to large-scale use for genetic containment.

## 1. Introduction

*LEAFY* (*LFY*) and its homologs across species are highly conserved in protein structure and play key roles in coordinating the transition from vegetative to reproductive growth, and in inflorescence patterning. *LFY* was initially characterized in *Arabidopsis*, where loss-of-function mutants (*lfy*) grew shoots or unusual flowers with leaf-like floral organs. Additional experiments demonstrated that *LFY* is key for floral meristem identity gene in *Arabidopsis*. *LFY* is typically present in a single copy but does have two homologs in some lineages (reviewed in [1,2]). The origins of *LFY* itself appears to pre-date both seed plants and flowering plants as *LFY*-like genes are present in moss, ferns, liverworts, and hornworts, as well as plants that form multicellular embryos but not seeds (reviewed in [2]). In seedless plants, LFY function appears to be key for sporophyte cell division [2]. In gymnosperms, *LFY* and its related gene *NEEDLY* (*NLY*) are expressed in meristems and appear to be important for both vegetative and reproductive meristem establishment (reviewed in [2]). The later-evolved primary targets of *LFY* in seed and flowering plants, the MADS family, appear to have arisen via MADS family expansion during speciation as there are no known orthologs in bryophytes or ferns [2,3,4,5].

It appears that some aspects of early *LFY* function have been retained in angiosperms outside of floral determination and patterning. For example, many herbaceous flowering plants show low levels of *LFY* expression in a range of vegetative tissues. *NFL*, the *LFY* homolog from *Nicotiana tabacum* (tobacco), is expressed in vegetative meristems [6]. Similarly, *FA*, the *Lycopersicon esculentum* (tomato) homolog is expressed in vegetative meristems, leaf primordia, and leaves [7]. In *Fabales* (pea), *PEAFLO* is an *LFY* homolog expressed in leaves [8]. This trend of *LFY* expression beyond floral tissues holds true for the very small number of studies of *LFY* in woody perennials. In several species of *Cornus* (dogwood) trees, the *LFY* homolog is present in leaves and bracts, with an expression pattern that appears to relate to the architecture of the overall inflorescence [1]. Poplar trees, the focus of this paper, have *LFY* expression in floral buds, flowers, vegetative buds, and leaf primordia [9].

Overexpression of *LFY* leads to strong alterations not only in flowers, but also in overall plant architecture. For example, 35S-*NFL1* transformed tobacco plants (tobacco *LFY* homolog) grew a single terminal flower and also had reduced stature [10]. Similarly, expression of 35S-*LFY* in hybrid poplar led to a bushy stature and small leaves [9]. While overexpression analysis of function can create de novo phenotypes it is also a useful means for analyzing gene function. In the case of LFY, overexpression in *Arabidopsis* leads to conversion of vegetative meristems to floral meristems and more rapid flowering [2,11,12,13]. These findings demonstrate the key role of *LFY* in floral meristem identity.

Information on functions of *LFY* in non-flowering plants is hampered by a paucity of loss-of-function mutants. Studies in *Physcomitrella patens* (moss) found that *PpLFY* double nulls show normal gametophytes, but the sporophyte either fails to develop after fertilization or has severe growth defects [14]. These data imply a key role of *LFY* in vegetative growth in the diploid life stage. Most loss-of-function studies of *LFY* are from examination of flowering plants, and focus on floral alterations, which are striking in nulls. There are very limited studies of the vegetative features of loss-of-function mutants of floral development genes, most studies are in herbaceous annuals, and none analyzed productivity effects in a randomized trial. For example, a novel *lfy* mutant in *A. thaliana* ecotype No-0 showed increased stem height and additional branches [15]. Similarly, tomato *fa* mutants (loss of *FLORICAULA*, tomato homology of *LFY*) leads to shoots growing in place of flowers, giving plants a bushy appearance [7]. Loss of the *LFY* homolog from peas gave alterations in leaf shape, with the formation of single rather than compound leaves, which is a dramatic alteration in overall leaf architecture [8]. Apart from the work of Klocko et al. 2016 [16], which is closely related to the present study, we are unaware of randomized or field-based studies of vegetative characteristics in *LFY* mutants or transgenics.

One potential application of loss-of-function *LFY* mutants is for prevention of unwanted gene flow by changing reproductive floral whorls into vegetative structures [16,17]. However, this usage would be limited to situations where the desired plant product is a vegetative structure such as a woody stem, and not a seed or fruit. Such applications are mostly likely when trees are grown for bioenergy, pulp, or wood products [17]. In such cases, it will be essential to determine if loss of *LFY* leads to alterations in plant survival, performance, or vegetative traits. Previously, we studied suppression of *LFY* in a large field trial of poplar trees [16], which included many constructs designed to alter the function of a variety of floral genes [18]. Of these, the strongly suppressed RNAi-*LFY* events gave strong alteration in floral form, leading to sterile female flowers, but statistically significant differences in tree growth were not detected. Due to the large-scale nature of the test (many constructs and gene insertion events, only a few replicates per event), and that there were only two *LFY*-suppressed sterile events observed, the power of this finding is low. We therefore vegetatively propagated several representative events from the original trial to create a much more highly replicated field trial with more statistical power to detect event differences if they are present. We report that although the trees appeared largely identical in vegetative form, the test had sufficient power to detect some differences in vegetative traits, mainly reductions in productivity traits including stem growth and leaf size that are of great interest for forestry applications.

## 2. Results

### 2.1. Most Events Rooted and Survived Well in Greenhouse Conditions

Events were selected from the initial large-scale trial and re-numbered to simplify naming (Table S1). The transgenic sterile (TS) events were selected because they had the strongest sterility phenotypes in the initial trial; transgenic normal (TN) events were selected based on the presence of normal catkins in the initial trial. Cuttings for establishing the new trial were rooted in a greenhouse and survival of cuttings by event were scored weekly. Of the 304 cuttings placed in the greenhouse, 262 survived for an overall survival rate of 86%. Cuttings of control trees had an overall survival of 89%, and all but one transgenic event had a survival rate of at least 91% (Figure S1). Event TN4 had a survival rate of 25%, and of the 10 surviving TN4 cuttings, 9 were from 1 parent tree. Due to its low rooting rate, and subsequent poor viability and growth in field conditions, it was excluded from statistical analysis.

### 2.2. Floral Traits

**Figure 1:**
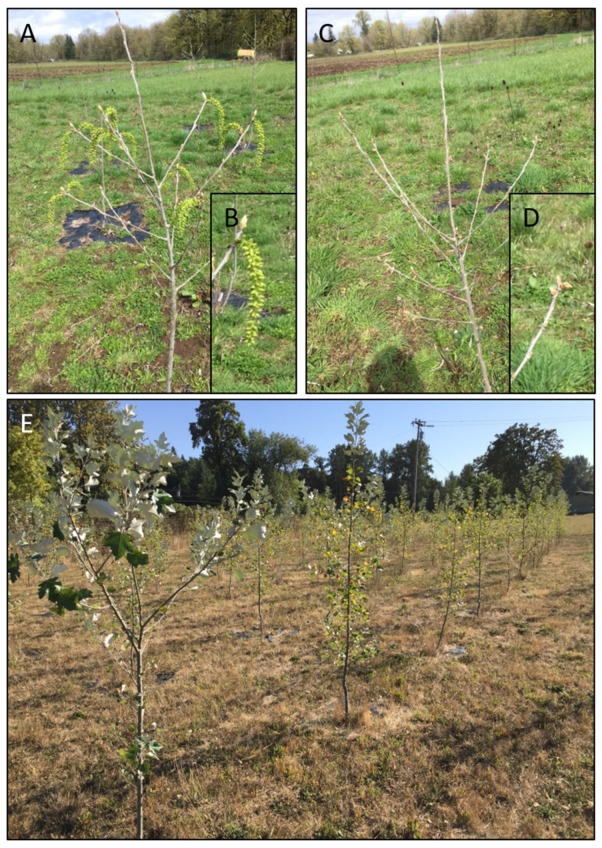
Field site overviews from 2019. (**A**) In spring 2019, WT trees had numerous large catkins, (**B**) WT catkins were fully open prior to leaf bud flush. (**C**) Sterile RNAi-*LFY* trees had numerous tiny catkins that opened late, (**D**) tiny catkins were still mostly enclosed by bud scales prior to leaf bud flush. (**E**) Field site overview in summer 2019.

Rooted cuttings were planted in July of 2016 in four randomized field blocks (Figure S2). Presence or absence of flowering was scored for all trees every spring. Trees with at least one floral bud or catkin were scored as flowering. In spring 2017, 3 of the 221 total trees flowered (1.4% of all trees including borders). These were one control tree (hereafter C for control), one transgenic normal tree (hereafter TN for transgenic normal), and one transgenic sterile tree (hereafter TS, Figure S3). All flowering trees were located in interior (non-border) blocks for an overall flowering of 2.1% of the 140 total interior trees. No flowering for any trees occurred in 2018. In 2019, 121 of the 132 interior trees under study (140 total trees minus 8 event TN4 trees) flowered (92%, Figure S4). Flowering across blocks ranged from 83–94% of trees, and flowering within events was 85–100% of trees, with event TS2 showing the lowest percentage of flowering trees. While flowering was similar across groups, blocks and events, there were differences in floral form and in the timing of floral bud flush. While control trees had large catkins which flushed prior to leaf flush, the TS events had small floral buds that flushed late (Figure 1).

**Figure 2:**
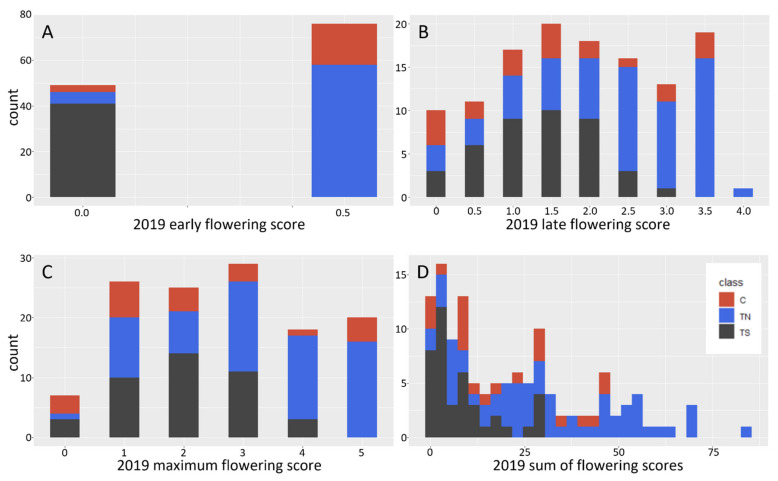
Floral timing and abundance varied among genotype classes, in all panels C is non-transgenic control (shown in red), TN is transgenic normal (shown in blue), TS is transgenic sterile (shown in dark grey). A numeric scoring system was used to score the timing of floral opening for all trees, with 0 no open buds, and a 0.5 to 5.0 relative scale for the number of open buds. (**A**) The first two weeks of the 2019 floral bud flush were designated “early flowering.” Trees with scores of 0 had no open flower buds, trees with scores of 0.5 had 10 or fewer open catkins. (**B**) Weeks 3–7 of the 2019 floral bud flush were designated “late flowering.” Here, a relative scale from 0–4 was used to score the number of open catkins (details in methods). (**C**) The maximal flowering score (number of open catkins) was determined for all trees. The distribution of this score was similar for C and TN groups, but still reduced for TS trees. (**D**) The metric “sum of flowering scores” is the actual counted number of open flowers per tree, TS trees tended to have the lowest scores, likely due to their delayed floral bud flush as seen in (**A**).

**Figure 3:**
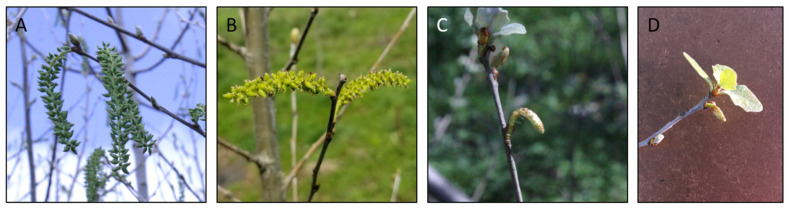
Catkin phenotypes were stable between the two field trials. (**A**) Catkins of control trees from the initial field trial in 2015 and (**B**) control trees from the new trial in 2019 were similar in appearance, as were (**C**) diminutive catkins of transgenic sterile trees from the initial trial in 2015 and (**D**) transgenic sterile catkins from the new trial in 2019.

To quantify the timing of floral opening, floral bud burst was scored weekly for 7 weeks, a 0–5.0 scale was used to score the number of open floral buds (details in methods). Scores from the first two weeks were used for the early flowering score, weeks 3–7 were used for the late flowering score. Analysis of floral timing, early (first two weeks) vs. late (last 5 weeks), of the growing season showed that both TS events had highly delayed floral opening, with no open floral buds observed in the early part of the flowering season (Figure 2). By contrast, most flowering TN and C trees had open flower buds in the first two weeks of floral timing scoring. Chi-squared analysis of early and late flowering scores showed TS trees were highly significantly different than TN or C trees (*p* < 0.001, Table S2. These floral buds did open, but the maximal floral score (the highest floral score given to that tree for the entire season) and sum of flowering scores (number of open flower buds) were lower for TS trees than for TN or control trees (*p* < 0.001, Table S2, Figure 2). Control trees and TN trees had similar floral timing, with most trees having open flowers in the early part of the floral season. Control and TN trees had similar maximal floral scores, and some TN trees had higher overall floral scores than control trees (Figure 2). Observations of floral form showed that two events, TS1 and TS2, had small catkins lacking externally visible carpels, similar to what was observed on these same events in the initial field test (Figure 3).

### 2.3. Vegetative Traits

Vegetative traits were measured for four years, starting in 2016 with size at planting. These 2016 data were excluded from overall analysis as field trees were coppiced in the first dormant season and had not fully and uniformly resumed growth. Data collected in 2017, 2018, and 2019 included wood volume productivity (calculated as volume index: height × DBH^2^) and leaf characteristics, including leaf area, petiole dimensions, and leaf-specific weight. In 2019 we also scored the timing of vegetative leaf flush, with both the top and bottom of each tree assessed separately. Chi-squared analysis of leaf flush indicated that top of tree flush was significantly different between groups (*p* < 0.01), but bottom of tree flush was not significantly different between groups (*p* = 0.5, Table S2). Initial principal components (PC) analysis of data from the 2019 growing season showed that block was a significant factor for every vegetative trait except for leaf specific weight (Table 1).

**Figure 4:**
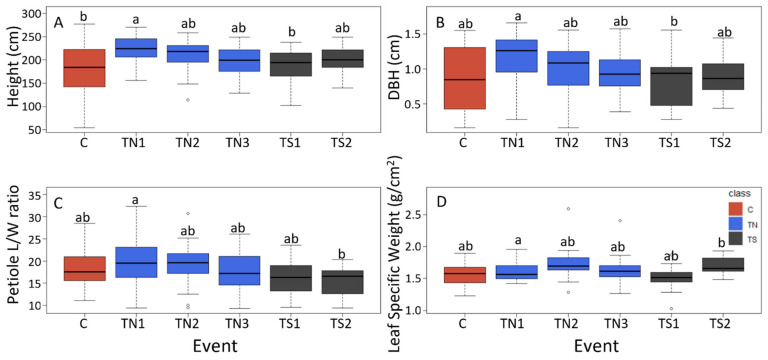
Representative traits with statistically significant variation among events or groups based on ANOVA for 2019. These included (**A**) height, (**B**) DBH, (**C**) petiole length to width ratio, and (**D**) leaf-specific weight. To remove block effects when visualizing event-based variation, boxplots were calculated based on deviations from block means for individual trees (with overall means added back to provided normalized trait values for each tree). Dark lines in bars show means of events, whiskers show extent of variation. Small letters indicate significant differences between events based on Tukey tests (*p* < 0.05).

Therefore, we included blocks in our Analysis of Variance (ANOVA) models and performed block adjustments (deviations from block means) when displaying genetic differences in phenotypes graphically. ANOVA showed significant differences within height, diameter, volume index, petiole L/W ratio and leaf-specific weight (Table 1, Figure 4). When comparing the groups of trees, significant differences in the 2019 data between TS and control trees included smaller petiole width of TS trees than C trees (*p* = 0.032). Significant differences between TN and control trees included larger height of TN (*p* = 0.003), and larger petiole width of TN (*p* = 0.041). Significant differences in productivity traits between the two sets of RNAi trees included DBH (*p* = 0.045) and volume index (*p* = 0.035) with TS trees being smaller than TN trees. Significant leaf trait differences between the two groups of RNAi trees (TS and TN) included petiole length with TS smaller than TN (*p* = 0.010), smaller leaf area of TS (*p* = 0.035), and lower leaf weight of TS (*p* = 0.035).

There was significant variation between transgenic (TN and TS) and control (C) genotypes, and between individual events, in vegetative traits measured in 2019 (Figure 4, Table S3). Controls as a group were highly variable in height and diameter compared to the transgenic events. Event TN1 tended to have larger trees, with larger heights and diameters than both control and TS trees. Statistically significant differences included height with TN1 trees larger than both C and TS1 trees. The DBH of TN1 was larger than that of TS1. Event TN1 also differed from TS events in regard to leaf traits; the petiole length to width ratio between event TN1 and TS2 differed with TN larger than TS2, and TN1 with a larger leaf-specific weight than TS2. Vegetative traits with no event-to-event differences in 2019 included volume index, petiole length, petiole width, leaf weight, and leaf area (Figure S5. The most variable vegetative trait was volume, a key productivity trait, in which event TN1 was 30.6% larger than the average, and event TS1 was 21.4% smaller than the event average (Table S3). The least variable vegetative trait was petiole width, in which C trees were 8.4% smaller than the event average, and event TN3 was 4.1% larger than the event average (Table S3). The largest differences in 2019 data were observed in the floral traits of TS events, with events TS1 and TS2 showing 100% and 99% reductions in early flowering as compared to the mean of all events (Table S3). Both TS events had reduced sum of floral scores −43% and −77% for TS1 and TS2, respectively (Table S3).

### 2.4. Principal Component Traits

**Figure 5:**
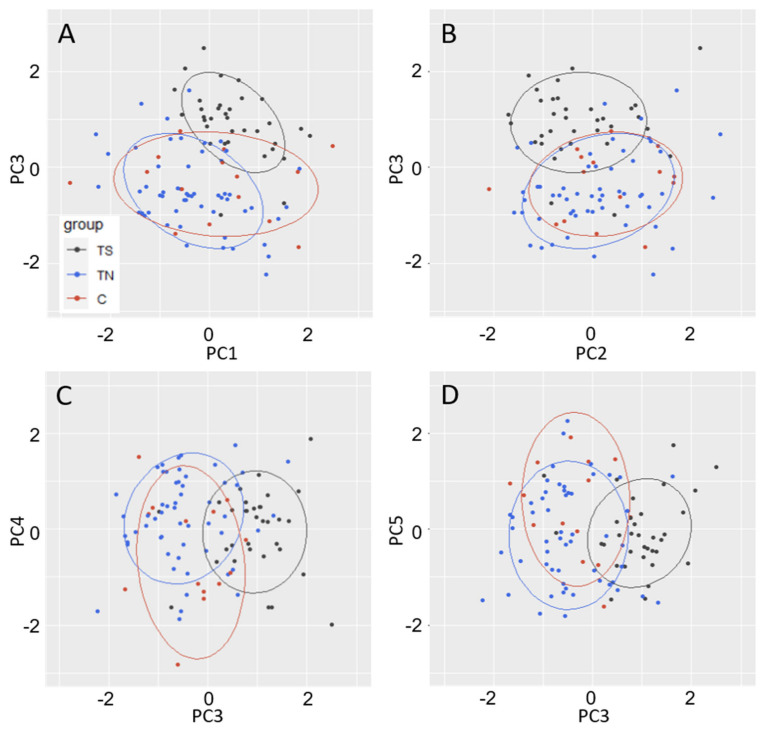
Elliptical plots of PCs where TS trees were statistically different (*p* < 0.05) from both TN and C trees based on Tukey comparisons from ANOVA. These included (**A**) PC1 and PC3, (**B**) PC2 and PC3, (**C**) PC3 and PC4, and (**D**) PC3 and PC5. Dots represent individual trees; ellipses show boundaries encompassing 65% of all data points.

Principal components analysis (adjusted for block) of floral characteristics in 2019, and of vegetative performance for all three years of field data from 2017–2019, showed that 68.5% of the variance could be described by 5 components. As is evident from the heat maps (Figure S6) and loadings summary table (Table 2), the PCs represented groups of traits, or traits in various years, but often in a complex manner. PC1, which accounted for 30.5% of the variance, clearly represented overall tree growth across years. PC2, which accounted for 13.2% of the variance, was a complex combination of petiole and leaf traits between years. PC3, which accounted for 9.1% of the variance, was most strongly affected by flowering traits. PC4, which accounted for 8.3% of the variance, was a complex combination of flowering, and leaf traits. PC5, which accounted for 7.4% of the variance, was also a mix of leaf traits, leaf flush, and flowering timing. The main traits of influence (absolute value of 0.2 or more) for PC1 were growth related, while the main traits in PC3 were floral related (floral timing, intensity, Table 2). These traits in PC4 were leaf and petiole related (leaf weight, petiole dimensions, Table 2), and for PC5, they were leaf and petiole related (leaf-specific weight, petiole length). There were statistically significant differences between events for three of the top five PCs (not PC2 and PC4: Table 1). Tukey contrasts showed that TN and C trees were significantly different in PC4 (*p* = 0.006) and PC5 (*p* = 0.023). TN and TS trees were significantly different in PC1 (*p* = 0.001), the productivity component, with TS being substantially lower than TN (Table 2; note that the productivity trait loadings are all negative). PC3, the main flowering PC, was also significantly different between TN and TS (*p* < 0.001), with TS being substantially lower than TN (Table 2; floral activity loadings all negative). TS and C trees were also significantly different for PC3 (*p* < 0.001). Histograms of PC outputs provided another way to visualize results. TS trees tended to be different in performance from TN and C trees in the directions predicted by the contrasts. This is most evident for PC1, PC2, and PC3. PC3, which is mainly related to floral traits, shows TS most distinctly (Figure S7, Table 2). Elliptical plots comparing PCs to each other also showed that TS trees were distinct from TN and C trees in regard to PC3 as plotted against all other PCs, though the statistical difference of TS from TN and C are also evident (Figure 5 and Figure S8). These plots also illustrate the wide range of performance of the C trees for some PCs. Block adjustment clearly helped to resolve the differences of TS trees from others in elliptical plots (Figure S9). The floral timing and intensity differences in the PCs were aligned with our analysis of this trait alone, with TS trees showing fewer and late-opening catkins. ANOVA for PC1, the PC which was well-loaded with the growth traits height, DBH, and volume for multiple years, had statistically significant variation among events and between TS and TN (Table 1 and Table 2). The many leaf and petiole morphology traits, contrasted among years as loaded into PC2, were not statistically significant among events or groups (Table 1). PC3, mainly affected by flowering traits, were highly significant among events and TS trees were significantly different from both TS and C groups (*p* < 0.001). PC4, which was mainly loaded with leaf morphology traits, and significantly differentiated TS and C but not other groups.

## 3. Discussion

An important aspect of this study was to assess the stability of floral phenotypes of sterile RNAi-*LFY* trees after propagation through vegetative cuttings. As these trees are female-sterile, they could not be propagated through seeds. Additionally, plantation trees are generally established with cuttings, either rooted or dormant, or via grafted plants, rather than seeds (for example, see [19,20,21]). We noted early on that one event, TN4, performed poorly in both greenhouse and field conditions. In hindsight, this event should have been excluded from the field planting. However, this event showed no indication of such poor performance in the initial field trial testing of RNAi-*LFY* events [16]. It is possible that the cuttings used for propagating these samples were in poor health. Of the 40 initial cuttings for this event, only 10 rooted in greenhouse conditions, and all but 1 of these rooted cuttings were from the same parent tree. It is also possible that there was tree-to-tree variation in rooting ability, as rooting of mature tree cuttings is known to be challenging and variable due to physiological differences in vigor and location/maturity of cuttings [22].

We found that the use of mature poplar trees for cuttings initially lead to maintained maturity (early flowering), as we observed flowering for three trees in 2017, the first spring after planting. However, all trees reverted to a more juvenile state the next year, with no trees flowering in 2018. The rooted cuttings apparently underwent a developmental reset to reenter the juvenile phase, thus observed phenotypes were not simply due to carryover of parental tree performance. This is commonly seen in poplar plantations, even when large trees or hedges are used as sources of cuttings [23]. In 2019 most trees of this precocious genotype flowered (91.7%), giving us a good view of floral form, timing, and abundance. Importantly, we found that floral phenotypes were stable between the initial field trial and this new one established with cuttings, with catkins of our two sterile events being smaller than control catkins with no externally visible carpels. Therefore, it is possible to see continued sterility after propagation with RNAi as the molecular approach to achieve gene suppression and genetic containment. As with most other studies of transgenic forest trees, the traits imparted were highly stable under vegetative growth and propagation [24] including those with a focus in suppression of floral genes such as *AGAMOUS* in poplar [25]. However, to our knowledge, this is the first observation of stability of floral sterility after vegetative propagation.

Analysis of floral form and timing showed that the TS trees showed delayed floral bud burst and small catkins, which is consistent with their initial characterization in the large-scale screening trial. The TS trees also had a lower maximal flowering score (the highest score of open floral buds achieved), and a lower sum of floral scores (the number of open catkins per tree in 2019). However, these two metrics were a bit challenging to assess as the floral buds for the TS trees were both smaller and opened later than those of C and TN trees, and the TS catkins emerged during vegetative bud flush.

These results were supported by the highly significant PC3, which was strongly influenced by floral traits. PC3 was significantly different between TS and control trees, and the main loadings for PC3 were related to leaf weight, petiole ratios, as well as several flowering traits. In general, the TS trees had late floral opening, lower floral abundance, and a change in the relative petiole dimensions as compared to control trees. These first two traits were easy to assess and quantify in the field, while the changes in leaves and petioles were more subtle and were only revealed during data analysis. TS and TN also showed significant differences in PC3, indicative of the differences in their floral abundance and timing of floral opening. This late floral bud opening may indicate a more vegetative-like identity of the floral buds, which would be consistent with a partial conversion of reproductive structures to vegetative structures. These data also show we were able to successfully maintain female tree sterility after vegetative propagation, as discussed above.

We found that block was a significant factor for performance of nearly all vegetative traits. Trees were initially planted in blocks by size (largest trees in block 1, smallest trees in block 4), which probably gave a growth advantage to the larger trees. Additionally, there was variation present from the environment, as this growing site was not uniform soil quality. There were also confounding factors including deer browse, which occurred unevenly across the site prior to mitigation of deer intrusion (browsing was mainly in block 4, which also had the smallest trees at planting). Block adjustments to data allowed for better resolution of differences between tree groups, as can be seen by the clearer separation of TS trees from TN and C in elliptical plots of PC2 (mainly relating to leaf traits) and PC3 (mainly relating to floral timing and abundance).

We did not observe any major alterations in overall tree architecture or form between our RNAi trees and our control trees. Unsurprisingly, this is in contrast to findings in the annual plant *Arabidopsis*, where analysis of a novel *lfy* mutant showed very noticeable impacts on vegetative features, such as increased number of rosette leaves, additional stem-born branches, and longer stems related to delayed onset and structure of flowers [15]. Annual *Arabidopsis* plants normally grow several terminal floral structures along a main stem, while the *lfy* mutants of *Arabidopsis* have branches in these equivalent positions [15]. By contrast, floral development in perennial poplar starts to form floral buds the growing season prior to floral bud flush, and these buds remain dormant over the winter [26]. Most floral buds occur around the outermost branches of the crown but can be found on the apex of the main shoot as well [27]. Thus, floral buds are formed in different places on the plant in poplar as compared to *Arabidopsis* and poplar are kept in a dormant state until the next growing season. One vegetative difference was the timing of leaf flush at the tops of trees, with TS trees flushing later than control and TN trees, which was a loading of PC5. Given that the floral buds of TS trees also flushed late, it may be that *LFY* has a role in the timing of overall bud flush.

We chose to use normal flowering events as a transgenic control for our study as these trees shared a transformation and propagation history with the transgenic sterile trees. These trees were chosen based on morphologically normal catkins in the initial field trial rather than on levels of *LFY* suppression as we did not previously characterize expression levels of all RNAi events. We observed significant differences between the overall tree size of some TN and TS events, with event TS1 being significantly shorter in height and smaller in DBH than event TN1. Event TS1 had the smallest height of all events, while TN1 was the largest event based on height, and was larger on average than non-transgenic controls as well. As events represent individual genetic transformation events with the construct insertion into a different place in the genome, it is expected to generate some event-to-event differences. However, these differences occurred both within a normal flowering event (TN1), indicating little impairment of *LFY* function, and events where flowers are sterile (TS1), indicating strong impairment of *LFY* function. Thus, it seems unlikely these large growth differences are mainly a result of variation in *LFY* expression, however, that was not characterized in this study. Moreover, there was wide variation within the wildtype control trees, a likely result of variation due to propagation timing, plant history on the site, and soil fertility. Nevertheless, our ANOVA results showed that the TS group was significantly different than the TN group for several traits, all of them indicating slower growth rate and smaller leaves in TS vs. TN trees. The productivity difference for volume growth exceeded 25%, a very large potential reduction in productivity. This result was also supported by ANOVA for PC1, the “productivity PC” as discussed under results, where its values were highly significantly different for TS vs. TN in the same direction. Significant differences were also observed in petiole traits, which in poplar are linked to tree growth as changes in petiole size is linked to leaf exposure to light [28]. In poplars, petiole size not only varies between clones, but also within the canopy of one tree, making it a very plastic trait [28]. It may be that the size differences observed between some TN and TS events could be due to both direct vegetative roles of *LFY*, and indirectly due to alterations in light capture ability as influenced by the petiole architecture.

Given the large within group and event-to-event variation, a much larger number of transgenic sterile and normal events would have been desirable for this study; there were just two sterile and three normal RNAi events, and event TN1 tended to have the largest trees, with the tallest height and largest diameter of all events (Figure 4), perhaps indicating an overall health difference of this event. Unfortunately, only two events with sufficient *LFY* suppression for these studies were discovered from our earlier studies [16] limiting our scope of inference.

In sum, our findings support a role for *LFY* function in vegetative features and performance of poplar trees, as has been observed for other angiosperms. Despite considerable variation among and within events, the weight of evidence suggests that *LFY* suppression is not without deleterious vegetative consequences on productivity. Though our results showing stability of floral phenotypes after vegetative propagation are encouraging, the use of *LFY* suppression for genetic containment may come at a large cost. Before *LFY* suppression mutation is deployed for genetic containment, additional studies are needed in relevant genotypes and environments to examine if the productivity losses we observed apply, and if so, what their magnitude is. In addition, other approaches to containment that do not appear to have such costs, such as suppression of *AGAMOUS*, should continue to be pursued.

## 4. Materials and Methods

### 4.1. Plasmid Construction and Plant Transformation

In the initial field trial, the RNAi-LFY cassette was generated in the pHannibal vector using a 285-bp fragment of *PtLFY* from *Populus trichocarpa* using standard organogenic techniques (as described in [16,18]). This was inserted into *Populus alba* (*P. alba*) female 6K10 clones via a binary vector, pART27 using *Agrobacterium*-based transformation. Events were rooted and four ramets per event were produced to be grown in the initial field trial. This initial trial was planted in summer 2011 [18].

### 4.2. Propagation and Root Induction

Dormant branch cuttings for propagation were collected in March 2016 from the initial 6K10 sterility trial. We selected non-transgenic control trees (C), four RNAi-*LFY* events with normal flowers (TN events), and two RNAi-*LFY* events with sterile catkins (TS events) for the field trial (Table S1). Each event was given a simplified number, rather than the longer event name of the initial trial (Table S1). Two trees from each of these selected events were used as a source of dormant woody branch cuttings for the focused trial. These cuttings were hand collected from the ends of branches and were approximately 1 foot in length. They were placed into plastic bags and stored at 4° Fahrenheit for 10 days before preparing cuttings and inducing rooting. Branch cuttings were trimmed to 10 cm length with the base cut at a 45° angle. After each cutting was trimmed, it was immediately dipped in a 1:5 dilution of Dip ‘N’ Grow rooting hormone and planted in a 3-inch pot filled with moist potting soil. Rooting hormone was freshly diluted for each event. Dormant buds were removed from each cutting, retaining 1–2 vegetative buds at the apex of the cutting. Inverted plastic bags were placed over pots to maintain high humidity.

### 4.3. Tree Acclimatization

Potted cuttings were moved to a greenhouse and randomized in two blocks. A shade cloth was used to lower the light intensity. Daytime greenhouse temperatures were between 70- and 85-degrees F, and cuttings were provided with constant water from trays under the pots. Bud flush of the cuttings was measured weekly as an indicator of cutting health. After most cuttings had flushed, cuttings were slowly acclimated to the natural humidity by cutting corners off the inverted plastic bags and eventually removing them entirely over the course of about 4 weeks. The first corner was cut off in week 1, the second corner in week 2, then the top of the bag snipped about half open for week 3 and fully open for week 4. After week 4, bags were removed. Flushed cuttings were moved to a lathhouse (outdoor screened in greenhouse) for acclimatization to outdoor conditions prior to planting. Tree height was measured in the lathhouse and used as a guide for planting in field blocks by size.

### 4.4. Field Design

Following acclimatization in a lath house (open sided greenhouse), rooted cuttings were planted in the field in July of 2016 (Figure S1). The trial consisted of 4 randomized blocks, with a one-tree border between each block and a one-tree perimeter border, with 9–40 trees per event (Figure S2, Table S4). Blocks consisted of rectangular arrangements of 5 × 7 trees with a 1-tree border between and around each block. The total field measured 90 feet (27.4 m) by 120 feet (36.6 m). Trees were planted at seven foot (2.1 m) spacing, their bases were covered with shade cloths and metal tags were placed for identification. Block designation was based on tree size at planting with block 1 being the largest trees and 4 being the smallest trees. Within the blocks, approximately five trees of each event were planted with randomized locations. Border trees were randomly planted using the remaining trees and included trees from each event as well as controls. On the west exterior border of the field, pairs of each event were planted in a non-randomized order as a showcase for demonstrative purposes. The border trees and showcase trees were excluded from statistical analyses and results. A total of 221 trees were planted: 30 controls, 114 transgenic normal, and 77 transgenic sterile (Table S4).

This trial was established under permit from APHIS (BRS permit number 17-012-101r-a2, previous permit numbers 17-012-101r-a1, 17-012-101r, 13-330-102r and 10-260-102-a1). Prior to field planting in July 2016, the field was sprayed with herbicide, tilled, and irrigation lines were established. Sprinkler heads with 360° rotation were attached to the irrigation lines which were spread out in an E-shape to provide full coverage of the field trial. From July through August 2016, the field was watered for four hours each morning. In September 2016, fields were watered for two hours each morning. Trees were irrigated according to this plan in 2017 as well, then the irrigation system was removed in early 2018.

### 4.5. Vegetative Growth Measurements and Spring Leaf Flush Scoring

Tree height was measured at planting and again in each dormant season. Height was collected using a meter stick in the first season of growth and using cm tape in later growth seasons. Stem diameter was measured at 6 inches above soil level using Meba IP54 Electronic Digital Calipers. Volume index was calculated using height × DBH^2^. Spring leaf flush was scored for the top and bottom half of each tree using the following scoring system; 0 = no leaves flushed, 1 = buds starting to break, 2 = partial leaf emergence, 3 = leaves fully flushed. Tree survival was scored each spring along with the timing of leaf flush. Trees that did not flush leaves were scored as dead. Because event TN4 had poor performance in terms of growth and survival in the field, it was excluded from future analysis.

### 4.6. Leaf Collection

Each year, three leaves were collected and measured from each tree. Either in the field, or shortly after collection, chlorophyll content was measured using a hand-held SPAD 502 Plus Chlorophyll Meter. Three SPAD measurements taken and averaged on each leaf. During the first growth season, leaves were collected at the visual midpoint of the trees. For subsequent years, leaves were collected using a leaf plasticron index. This was measured by counting ten leaves down from the top of the tree without including nascent leaves. Nascent leaves were those under 4 cm in length. Leaves were collected from different sides of the tree when possible. Any trees with fewer than 10 developed leaves were not included in the statistical analyses. No chlorophyll content was measured in 2019.

### 4.7. Leaf Trait Measurements

Petiole length and width of fresh leaves were measured with digital calipers. Leaves were scanned using an HP Scanjet 8200. Each scan included three leaves from the same tree and a ruler for a scale marker. Leaf area in cm^2^ was calculated using ImageJ software. Scanned leaves were placed in drying ovens until the mass of the combined leaves remained stable, indicating leaves were fully dry. Each leaf was weighed with a Mettler AJ100 scale. Leaf-specific weight was calculated as the dry weight divided by the leaf area.

### 4.8. Scoring Flowering, Floral Timing and Floral Abundance

Trees were scored yearly for the presence of floral buds and fully flushed catkins. Trees with at least one floral bud were scored as flowering. In 2019 trees were scored for floral bud presence (flowering or not), timing of floral opening, and abundance of open flowers (sum of flowering scores). Floral opening was scored for 7 weeks beginning 11 March 2019 using the following system.

0. No fully flushed catkins.
1. Very sparse fully flushed catkins (less than 10).
2. Sparse fully flushed catkins (more than 10 but less than 20).
3. Some fully flushed catkins (more than 20 but less than 40).
4. Many fully flushed catkins (more than 40 but less than 50).
5. Abundant fully flushed catkins (50 or more).

Trees with numbers of fully flushed catkins that fell about halfway between two categories were scored at half integer intervals. Maximal flowering score was the highest overall score given to a tree across the 7 weeks.

Floral abundance was determined by counting the actual number of fully flushed catkins on each tree. This was performed twice, once on 25 March 2019, and once on April 4 2019. These data were termed “sum of floral scores.”

### 4.9. Field Management

In summer 2016 several of the trees experienced browsing damage from deer. Due to uneven tree performance in the 2016 growing season, it was decided to trim all trees to a uniform height of 30 cm from soil level. Trees that had not reached 30 cm in height were left untrimmed. Trees were trimmed during the first winter season then singled in spring 2017. Trees were terminated in fall 2020 by coppicing (cutting off near ground level), followed by injecting EZ-Ject copperhead herbicide shells containing imazapyr directly into the stem roughly 3 inches (7.6 cm) off the ground. Stems were incinerated while the stumps remained in the ground.

### 4.10. Data Analysis

All statistical analyses were performed in R studio, raw and analyzed data can be found in Ap raw R code can be found in Supplementary File S2. All trees that were recorded as dead in any year, even if they resprouted in another year, were excluded from the analysis due to poor growth, as were all data collected in 2016 due to deer damage and trimming.

Using all collected data from 2017–2019, a principal component analysis was performed using the function prcompfrom the stats package [29]. Prior to analysis, all rows containing missing data were omitted with the function na.omit from the stats package, and all data were adjusted for block. After adjusting for block, the polarity of PCA results changed, meaning the major growth traits are negative, and the more negative a trait is, the larger the tree is in reality. Package dyplr [30] was used to assign groups and events to their respective blocks using the function mutate. Linear models and ANOVAs were created with the principal components 1–5. All linear models were created using the function lm in the stats package. Linear models for the principal components were created with event as the independent variable.

Traits were independently analyzed within the 2019 collected data only. This is because after the third year of growth, the trees reached maturity and produced mature leaves and flowers. As such, data collected in 2019 were considered the most reliable data. The 2019 data were subset into 4 datasets to exclude missing data values without excluding whole rows. This was done using the function subset in the base R package [29]. All individual trait analysis was performed on raw data unadjusted for block. Linear models were created for each numerical variable within the 2019 collected data. All linear models were created with event and block as the independent variable. In all linear models, block × event interactions were ignored as they were found to only be significant in 2 variables.

Logistic regressions were created with event as the independent variable for each categorical variable within this dataset with the function glm in the package brglm2 [31]. Prior to analysis, categorical variables were collapsed into binary variables. All floral scores were collapsed from 0–2 (to become zero) and 3–5 (to become 1), while leaf flush scores were collapsed in the same manner from 0–1 and 2–3. The sum of floral scores over the whole season were collapsed from values greater than the median. All logistic regressions were tested for complete separation. Where complete separation was detected, penalized logistic regressions were created. Diagnostic Q-Q and residual plots created with package DHARMa [32] and function simulateResiduals indicated that assumptions of normality and equal variance were acceptable. Likelihood ratio tests were performed on all logistic regressions using the function drop1 from brglm2. Manual chi-squared tests were used to uncover differences between classes.

Diagnostic plots were created for all linear models, which found the assumptions of normality, equal variance, and independence to be acceptable. ANOVAs were then performed on all linear models using the function anova in the stats package. These were used to then perform Tukey’s HSD test as well as contrasts using the function emmeans in the package emmeans [33].

Graphics for all data were created with ggplot2 [34], ggbiplot [35] (dependency devtools, ggplot2), plotly [36], graphics in base R, and multcompView [37]. The color palette for all graphics is as follows: grey27, royal-blue, and tomato3. All boxplots display block adjusted data.

## Figures and Tables

**Table 1:** Summary of *p*-values from ANOVA tests and Tukey comparisons. Comparisons between groups are shown with a-sign, such as TS-TN. Significant values (*p* < 0.05) are shown in bold; NA is non-applicable.

ANOVA Results															
**Event** **Block**	**Result**	**Height**	**DBH**	**Volume Index**	**Petiole Length**	**Petiole Width**	**Petiole L/W** **Ratio**	**Leaf Area**	**Leaf weight**	**Leaf-Specific Weight**	**PC1**	**PC2**	**PC3**	**PC4**	**PC5**
	***p*** **values**	**0.002**	**0.026**	**0.036**	0.053	0.083	**0.015**	0.153	0.055	**0.003**	**0.002**	0.143	**<0.001**	0.054	0.095
	***p*** **values**	**0.002**	**0.004**	**0.001**	**<0.001**	**0.014**	**<0.001**	**<0.001**	**0.002**	0.295	NA	NA	NA	NA	NA
**Tukey Comparisons**															
**TS-TN**	***p*** **values**	0.102	**0.045**	**0.035**	**0.010**	0.943	**0.004**	**0.035**	**0.015**	0.344	**0.001**	0.211	**<0.001**	0.427	0.961
	**% change**	−7.67	−17.70	−26.47	−15.08	1.02	−15.75	−20.22	−23.95	−3.28	NA	NA	NA	NA	NA
**TS-C**	***p*** **values**	0.235	0.990	0.854	0.856	**0.032**	0.167	0.621	0.700	0.745	0.455	0.181	**<0.001**	0.097	0.056
	**% change**	−9.81	−1.79	−9.70	−4.09	−11.79	−12.75	−11.16	−10.65	−2.43	NA	NA	NA	NA	NA
**TN-C**	***p*** **values**	**0.003**	0.131	0.394	0.176	**0.041**	0.787	0.572	0.347	0.145	0.266	0.809	0.623	**0.006**	**0.023**
	**% change**	18.94	23.67	22.81	12.94	10.66	4.16	11.35	17.33	5.91	NA	NA	NA	NA	NA

**Table 2:** Summary of traits with large PC loadings. Traits shown are those with absolute values of 0.2 or greater for PC 1-5. Positive associates are those traits with a positive loading value, negative associations are those with a negative loading value. For simplicity, significant traits of the same type from different years have been combined into one cell, where numbers indicate collection year (e.g., 17 is 2017).

Loading Type	PC1	PC2	PC3	PC4	PC5
Positive associations	none	leaf weight 17 leaf area 17 petiole length 17 petiole width 17 petiole ratio 17	leaf weight 18	late flowering	leaf area 19 leaf weight 19 petiole length 19
Negative associations	leaf weight 19 petiole length 19 DBH 17, 18, 19 height 18, 19 volume 17, 19	leaf area 18 petiole width 18 leaf weight 18 SPAD 18 leaf-specific weight 18	early flowering late flowering max flowering intensity sum flower petiole ratio 17, 18	leaf area 17, 18 leaf weight 17, 18 leaf-specific weight 17 petiole width 17 petiole length 17, 18 max flowering intensity	petiole length 18 leaf area 18 flush bottom leaf weight 18 flush top max flowering intensity petiole ratio 19 petiole width 18 late flowering

## Data Availability

Data are contained within the article or Supplementary Material.

## Supplementary Materials

The following are available online at https://www.mdpi.com/article/10.3390/plants10081594/s1, Figure S1: event performance in the greenhouse and early field views. Figure S2: General planting map of the field trial. Figure S3: Catkins on field trees in spring 2017. Figure S4: Trees flowered abundantly in 2019. Figure S5: Tukey plots of traits without significant differences between events trees in 2019. Figure S6: Heat map of PCA loadings based on deviations from block means Figure S7: histograms of PC outputs. Figure S8: Elliptical plots of PCs without significant differences between TS and TN or TS and C trees. Figure S9: Example of PC plot with and without block adjustment to show how adjustment increased resolution of class differences. Table S1: Key to event IDs and short names. Table S2: Summary of p-values from likelihood ratio and Chi-squared analysis of flowering and leaf flush traits Table S3: Percent change between event means for block-adjusted 2019 field data. Table S4: Numbers of trees in each block, event, and group at time of planting in 2016. Supplementary File S1: Excel Spreadsheet of All Data. Supplementary File S2: Raw R Code.
