# Supplementary material for: RNAi Suppression of LEAFY Gives Stable Floral Sterility, and Reduced Growth Rate and Leaf Size, in Field-Grown Poplars
Source: Plants (Basel). 2021 Aug 3;10(8):1594. doi: 10.3390/plants10081594 (PMC8398303; doi:10.3390/plants10081594)
Supplement: Supplementary file 1 [file plants-10-01594-s001.zip › Supplementary figures and Tables v15b.pdf]

Supplementary figures and Tables

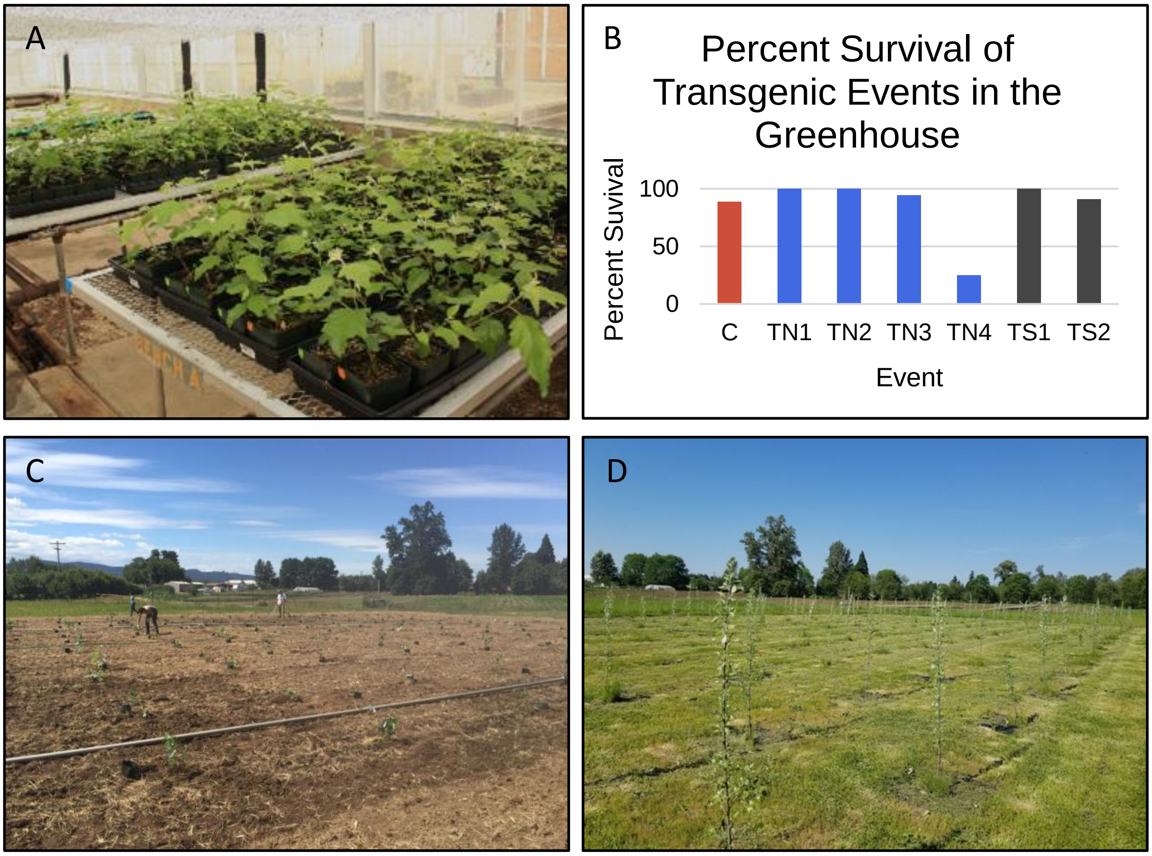

Figure S1: Event performance in the greenhouse and early field views
(A) Ramets were randomized into two blocks on two growth benches in the greenhouse to allow for rooting and shoot growth prior to field planting. (B) Ramet survival was monitored and used to calculate percent survival by event. (C) Trees were planted in the field in June 2016 with in-field irrigation. (D) In April 2018 trees were well established.

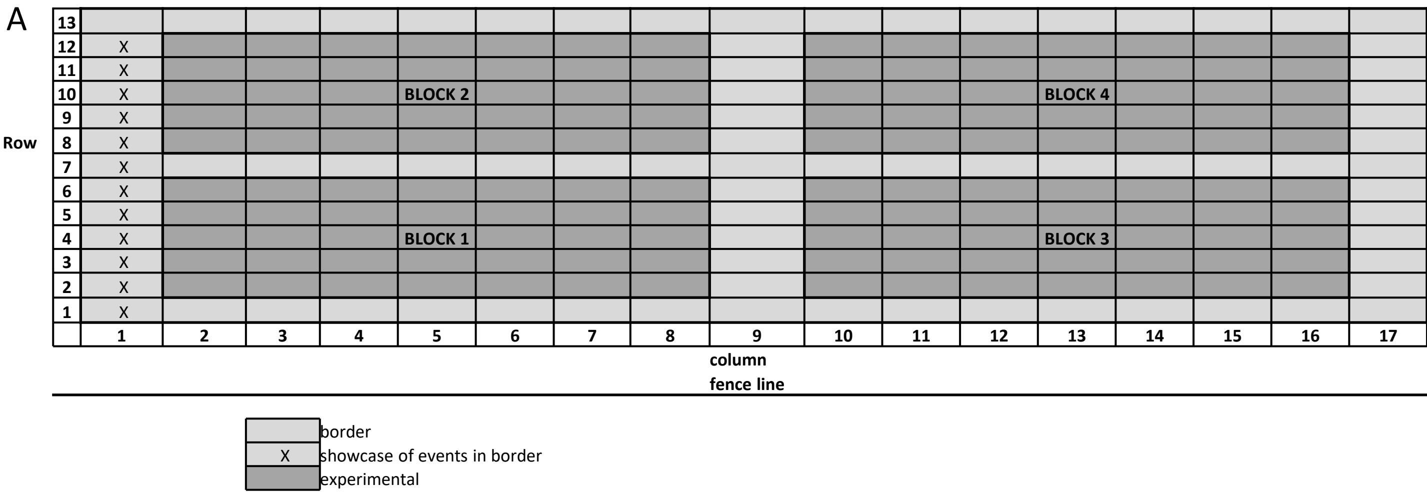

B

Row	1	2	3	4	5	6	7	8	9	10	11	12	13	14	15	16	17
13	B, TS2, 25	B, TN2, 31	B, C, 25	B, TN1, 33	B, TN2, 29	B, TN3, 23	B, TN3, 29	B, TS2, 35	B, TS1, 32	B, TN1, 32	B, TS2, 28	B, TS2, 33	B, TS2, 37	B, TN3, 21	B, TN2, 24	B, TS2, 34	B, TS1, 34
12	sh, TN3, 31	2, TN3, 7	2, TN1, 10	2, C, 6	2, TN3, 8	2, TS2, 6	2, TS1, 10	2, TS1, 7	B, TS2, 26	4, TN3, 19	4, TN2, 18	4, TN1, 22	4, TN1, 20	4, TS2, 19	4, TN3, 18	4, TN4, 10	B, TN1, 23
11	sh, TN3, 32	2, C, 7	2, C, 8	2, TN2, 10	2, TS2, 9	2, TS2, 11	2, TN2, 11	2, TN1, 7	B, C, 27	4, TN3, 17	4, TS1, 19	4, TN1, 19	4, C, 22	4, TS1, 21	4, TN3, 16	4, TN2, 19	B, TS2, 36
10	sh, TN2, 35	2, TN1, 8	2, TS2, 10	2, TN3, 9	2, TN1, 9	2, TN2, 7	2, TN2, 9	2, TS1, 8	B, TS1, 23	4, TN1, 18	4, TN2, 20	4, C, 31	4, TN4, 8	4, C, 21	4, TS2, 18	4, C, 19	B, TS1, 27
9	sh, TN2, 36	2, TS1, 9	2, C, 9	2, TN1, 11	2, TS1, 6	2, TN2, 8	2, TS2, 7	2, C, 10	B, TN1, 31	4, TS2, 20	4, TN3, 20	4, TS1, 20	4, TS1, 22	4, TS2, 40	4, TN4, 9	4, TS2, 21	B, TS2, 31
8	sh, TN1, 36	2, TS1, 11	2, TN1, 6	2, TN2, 6	2, TN3, 10	2, TN3, 6	2, C, 11	2, TS2, 8	B, TS1, 29	4, C, 20	4, TN1, 38	4, TN2, 21	4, TS1, 18	4, TS2, 22	4, C, 18	4, TN2, 22	B, TS2, 30
7	sh, TN1, 37	B, TN2, 30	B, TN2, 27	B, TS2, 24	B, TN1, 28	B, TN1, 25	B, TN3, 28	B, TN2, 25	B, TN1, 26	B, TS1, 33	B, TN2, 34	B, TS2, 23	B, TN1, 34	B, TN2, 28	B, TN2, 33	B, TN1, 27	B, TN1, 30
6	sh, TS2, 38	1, TN3, 2	1, TN2, 3	1, TN3, 4	1, TN3, 5	1, TS1, 3	1, TN3, 1	1, TS2, 4	B, TN2, 26	3, TN2, 15	3, TN3, 14	3, TS2, 13	3, TS1, 16	3, TN2, 14	3, TS2, 16	3, C, 15	B, TS1, 25
5	sh, TS2, 39	1, TS2, 3	1, TN2, 1	1, TN1, 4	1, TN2, 2	1, TN4, 1	1, C, 1	1, TN1, 2	B, TN4, 6	3, TN1, 16	3, TS1, 12	3, TN3, 13	3, TN1, 14	3, C, 16	3, TN1, 17	3, TN3, 15	B, TN3, 26
4	sh, TS1, 36	1, TN4, 5	1, C, 3	1, C, 5	1, TN1, 1	1, C, 2	1, TS2, 1	1, TS1, 5	B, TS1, 35	3, TS2, 15	3, TS1, 13	3, TS1, 17	3, C, 12	3, TS1, 15	3, TN2, 16	3, TN1, 15	B, TS1, 31
3	sh, TS1, 37	1, TS1, 2	1, TN4, 4	1, TS2, 2	1, TN1, 5	1, TN1, 3	1, TN2, 5	1, TN4, 2	B, TN3, 30	3, C, 17	3, TN1, 12	3, TN3, 12	3, TN3, 11	3, TS2, 12	3, C, 14	3, TS2, 17	B, TN2, 23
2	sh, C, 29	1, TN2, 4	1, TS1, 4	1, TS1, 1	1, TS2, 5	1, TN4, 3	1, TN3, 3	1, C, 4	B, TN3, 22	3, C, 13	3, TN2, 12	3, TS1, 14	3, TN1, 13	3, TN2, 13	3, TN2, 17	3, TS2, 14	B, TS1, 26
1	sh, C, 30	B, TS1, 24	B, TN2, 32	B, C, 24	B, TS2, 27	B, C, 28	B, TN1, 24	B, TN3, 25	B, TS1, 30	B, TN3, 24	B, TN1, 35	B, TS2, 32	B, C, 26	B, TS2, 29	B, TN1, 29	B, TS1, 28	B, TN3, 27
	1	2	3	4	5	6	7	8	9	10	11	12	13	14	15	16	17

Column

Figure S2: Planting maps of the field trial
(A) Placement of the 4 blocks of experimental trees (dark grey) and border trees (light grey), showcase events in the border (non-random) have an "X" through the box. (B) Specific map of the field trial showing control trees (blue), transgenic normal events (green), transgenic sterile events (orange), and border trees (light grey).

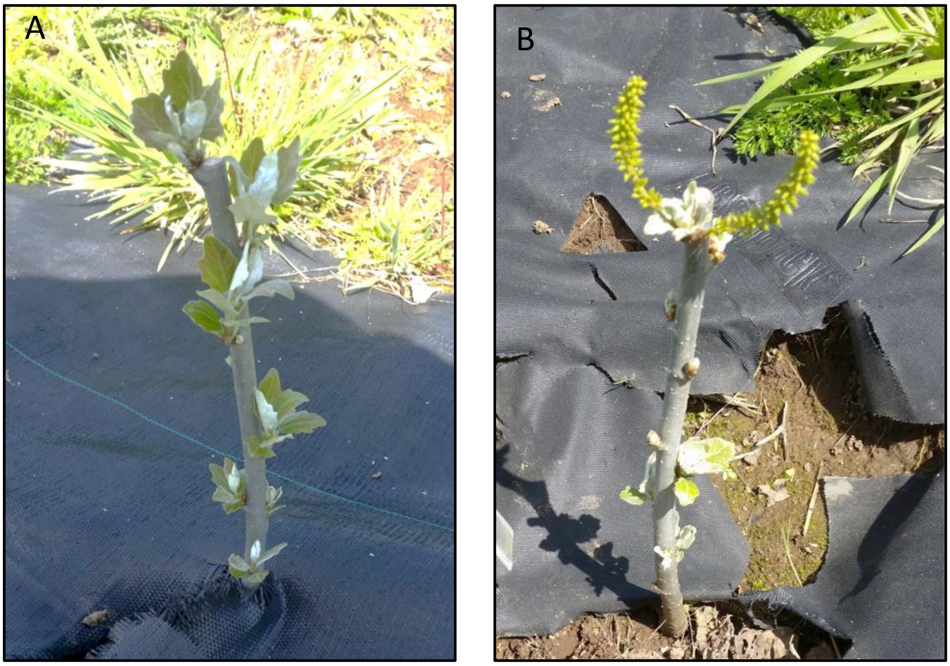

Figure S3: Catkins on field trees in spring 2017

(A) In spring 2017 most trees had vegetative leaves and no catkins (B) while three trees from different events had catkins, event TN2 is shown, with two large catkins originating near the cut apex of the stem.

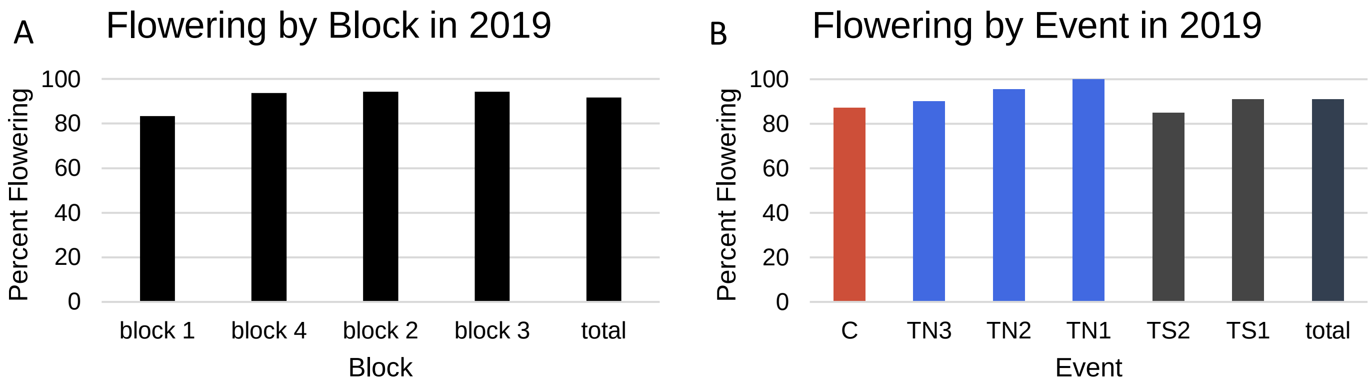

Figure S4: Trees flowered abundantly in 2019

(A) Trees in all four blocks showed abundant flowering in 2019, (B) as did all events.

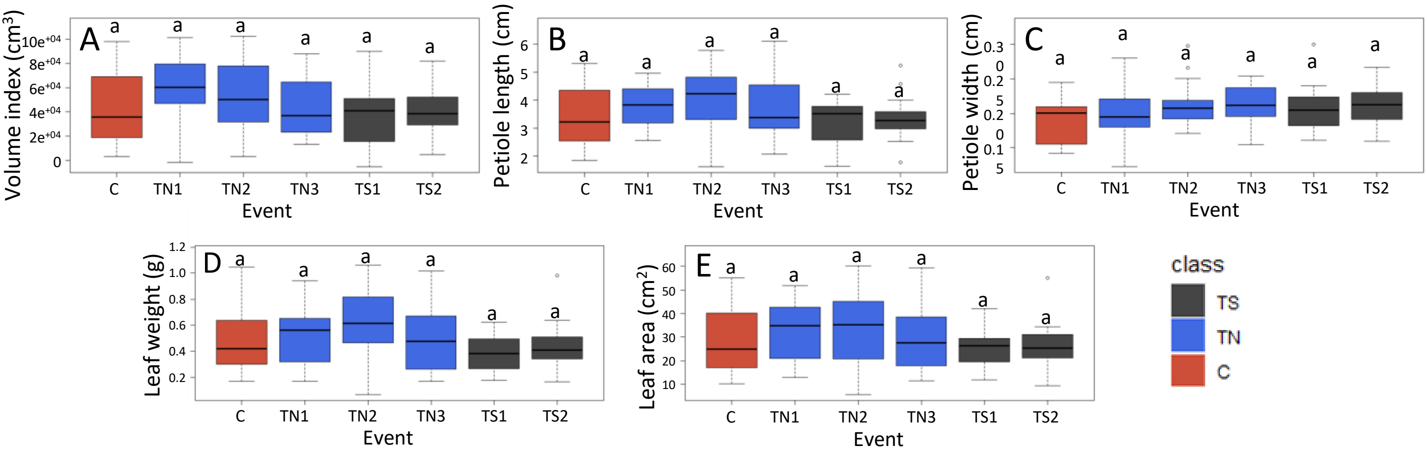

Figure S5: Tukey plots of traits without significant differences between events trees in 2019
No significant differences were observed for trees in 2019 for (A) stem volume index, (B) petiole length, (C) petiole width, (D) leaf weight, or (E) leaf area.

**trait**	**PC1**	**PC2**	**PC3**	**PC4**	**PC5**	**PC6**	**PC7**	**PC8**	**PC9**	**PC10**
% variance explained	30.5	13.2	9.1	8.3	7.4	4.3	3.8	3.1	2.9	2.8
early_flowering_b	-0.12272	0.047572	-0.38723	0.04899	-0.02579	0.019821	-0.09468	0.069753	-0.05448	0.09142
dbh_cm19_b	-0.24755	-0.02395	0.051041	0.180217	0.020252	-0.02629	-0.03965	-0.03045	0.131592	-0.24362
height_cm19_b	-0.27706	-0.03808	0.072734	0.080406	0.032705	-0.06643	-0.01994	-0.02639	0.147049	-0.09217
volume_cm19_b	-0.2655	-0.01808	0.041654	0.110521	0.035891	-0.03966	-0.04078	-0.03202	0.143369	-0.22219
flush_top_b	-0.09195	-0.09151	-0.18023	-0.00829	0.229957	0.489152	0.054608	0.248602	-0.05137	0.098891
flush_bottom_b	-0.0672	-0.14065	-0.12532	0.01535	0.258127	0.492193	0.155892	0.129344	0.087809	0.255501
max_flowering_intensity_b	-0.08324	0.069281	-0.33535	0.22316	-0.21428	-0.09184	0.206676	-0.25108	-0.08553	-0.0984
late_flowering_b	-0.09831	0.080133	-0.35105	0.215913	-0.20891	-0.03968	0.193048	-0.19516	-0.05713	-0.05869
sum_flower_b	-0.13599	-0.0155	-0.30618	0.184229	-0.06444	0.069609	0.165271	-0.05421	0.042924	0.141027
flower_binary_b	-0.04348	-0.03682	-0.19966	0.118618	-0.05324	0.101852	-0.09102	-0.34554	0.087958	0.305858
average_leaf_area19_b	-0.19333	-0.17452	-0.08101	-0.02918	0.312237	-0.18216	0.052019	0.056761	-0.11312	-0.1682
avg_petiole_length19_b	-0.20527	-0.17578	-0.06994	0.005644	0.290212	-0.21621	0.001939	0.024969	-0.10238	-0.03292
avg_petiole_width19_b	-0.04933	-0.12216	0.041733	-0.04855	0.123983	-0.37133	0.58854	0.10671	0.103297	0.164837
petiole_l_w_ratio19_b	-0.18144	-0.11559	-0.08508	0.033902	0.213432	0.050441	-0.4084	-0.01812	-0.1901	-0.17781
average_leaf_weight19_b	-0.20116	-0.17963	-0.06913	-0.00619	0.306243	-0.23219	-0.0144	0.033833	-0.06708	0.027505
leaf_specific_weight19_b	-0.08277	-0.08347	0.045072	0.079614	0.087309	-0.29117	-0.27556	-0.06977	0.10629	0.659938
dbh_cm17_b	-0.22029	0.091975	0.176029	0.099154	-0.19879	0.077324	-0.01733	0.176813	-0.14238	0.082911
height_cm17_b	-0.24298	0.072387	0.171191	0.114051	-0.08961	-0.02288	0.063215	0.065945	-0.12485	0.074445
volume_cm17_b	-0.23355	0.11139	0.181078	0.090821	-0.16604	0.063898	0.019574	0.118591	-0.12647	0.106556
average_leaf_area17_b	-0.13449	0.274103	-0.00505	-0.3396	0.089002	0.031257	0.061692	-0.13196	0.069748	0.067656
avg_petiole_length17_b	-0.17892	0.26727	-0.08619	-0.23781	0.065683	-0.00181	0.021004	-0.0752	-0.18691	0.005115
avg_petiole_width17_b	-0.16112	0.222985	0.048372	-0.24004	-0.00022	-0.01133	0.166619	0.01746	-0.22588	0.170723
petiole_l_w_ratio17_b	-0.12467	0.202766	-0.20608	-0.12106	0.106067	0.010985	-0.13877	-0.13125	-0.11013	-0.18332
average_leaf_weight17_b	-0.14292	0.282047	-0.02605	-0.31839	0.059337	0.007319	0.048533	-0.06975	-0.14082	0.089041
leaf_specific_weight17_b	0.041984	-0.14173	-0.00476	0.257131	-0.16777	-0.06377	-0.01843	0.287534	-0.63507	0.094818
spad_17_b	0.151543	0.078065	-0.09783	-0.14582	-0.03427	-0.20147	-0.12696	-0.01264	-0.227	0.078694
dbh_cm18_b	-0.23979	0.140289	0.181922	0.105455	-0.09987	0.063344	-0.05376	0.112145	0.036867	0.006675
height_cm18_b	-0.22237	0.143746	0.096454	0.158281	-0.00651	-0.0093	-0.01318	0.003854	0.214468	0.04123
volume_cm18_b	-0.24071	0.17616	0.152738	0.098887	-0.056	0.049175	-0.0292	0.054373	0.126593	0.030141
average_leaf_area18_b	-0.13819	-0.23054	-0.0693	-0.30126	-0.27252	0.008991	-0.0952	0.031665	-0.0088	-0.03268
avg_petiole_length18_b	-0.13411	-0.17179	-0.19814	-0.22156	-0.27694	-0.09495	-0.11105	0.248165	0.22124	0.028209
avg_petiole_width18_b	-0.149	-0.26121	-0.01693	-0.14864	-0.21032	0.000292	-0.18584	-0.13694	0.073427	0.109372
petiole_l_w_ratio18_b	0.006866	0.094211	-0.27233	-0.11394	-0.13512	-0.11881	0.07864	0.550229	0.217094	-0.09572
average_leaf_weight18_b	-0.14199	-0.2634	-0.01847	-0.28157	-0.2562	0.035857	-0.03818	-0.02037	-0.02039	-0.02678
leaf_specific_weight18_b	-0.09626	-0.28203	0.20178	-0.08374	-0.09317	0.115042	0.188286	-0.24571	-0.12193	-0.01785
spad_18_b	-0.10846	-0.27611	0.121552	-0.14062	-0.06396	0.162333	0.273524	-0.17467	-0.10703	-0.08634

Figure S6: Heat map of PCA loadings based on deviations from block means
Positive associations are shown in reds, negative associations are shown in blues.
Darker hues indicate higher absolute value loadings.

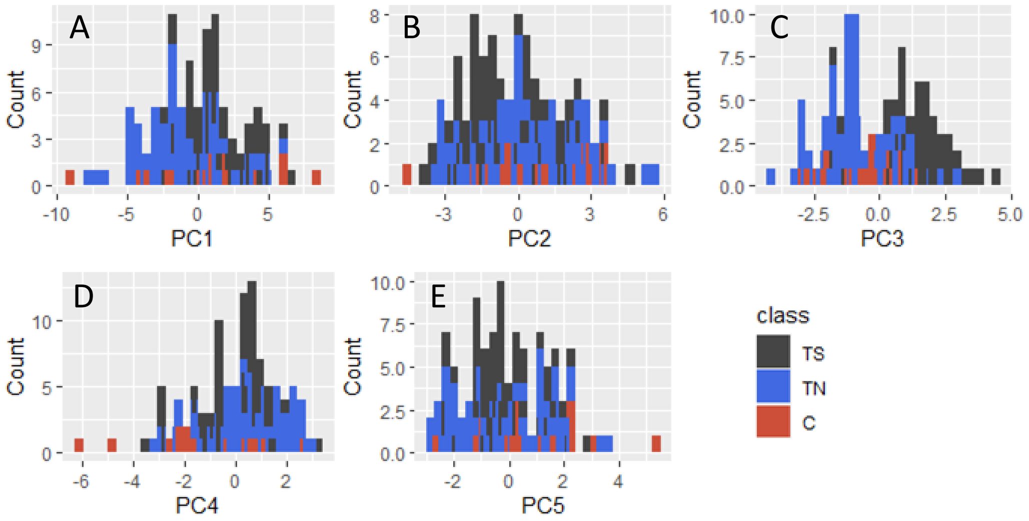

Figure S7: Histograms of PC outputs
Stacked bar plots of block adjusted data for (A) PC1, (B) PC2, (C) PC3, (D) PC4 and (E) PC5. Bar colors show class of events, control C in red, TN in blue, TS in grey.

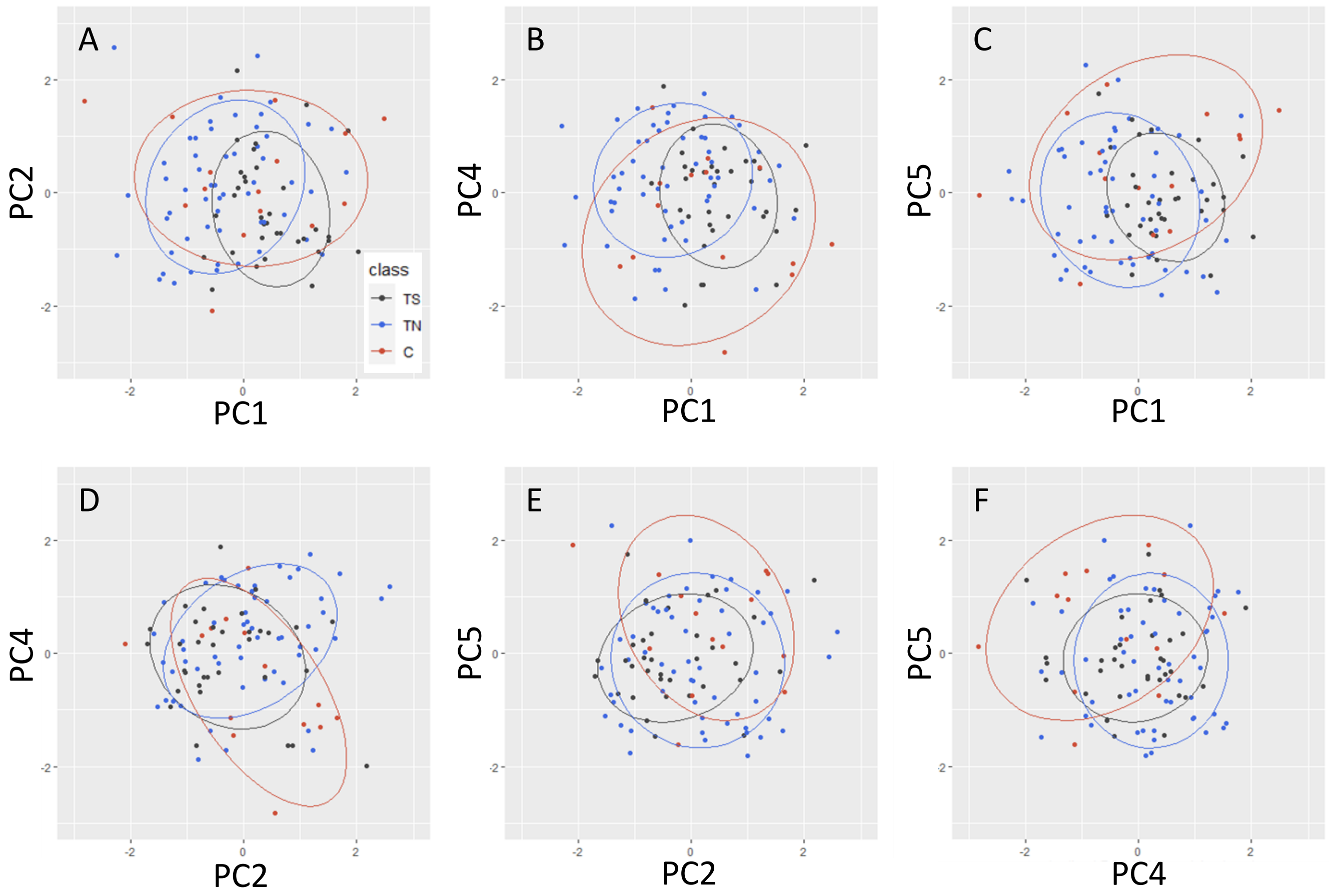

Figure S8: Elliptical plots of PCs without significant differences between TS and TN or TS and C trees
Comparisons of (A) PC1 and PC2, (B) PC1 and PC4, (C) PC1 and PC5, (D) PC2 and PC4, (E) PC2 and PC4, and (F) PC4 and PC5. Black circle is TS, blue is TS, red is C.

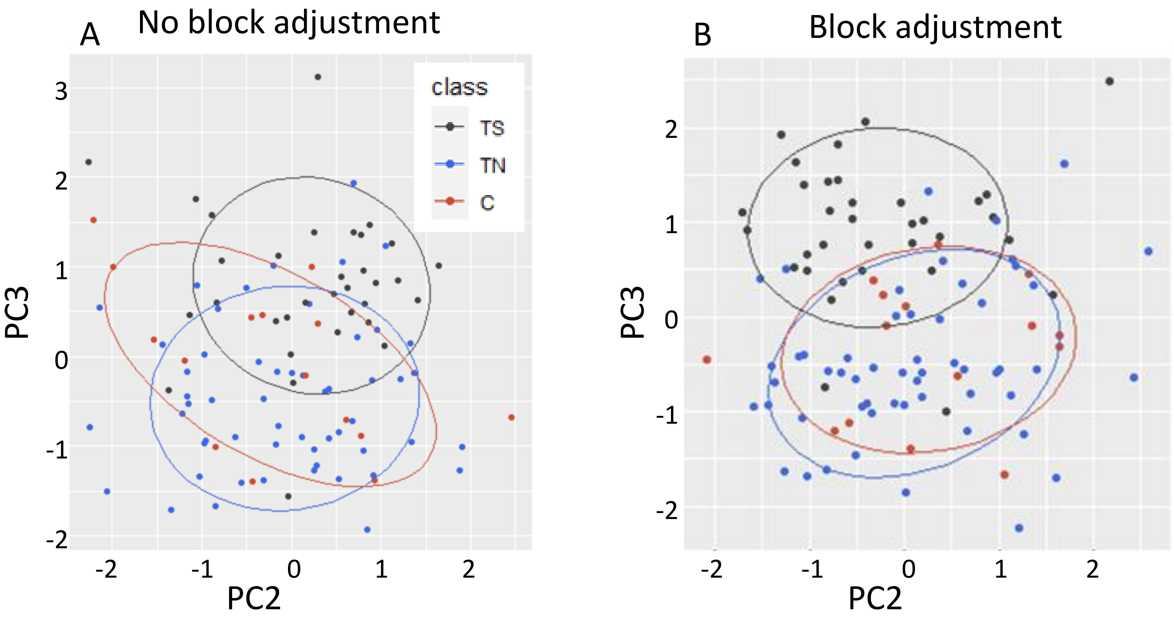

Figure S9: Example of PC plot with and without block adjustment to show how adjustment increased resolution of class differences

Elliptical plots of PC1 and PC3 (A) without block adjustments and (B) with block adjustments. PC3 main loadings are related to floral timing and abundance. PC 2 main loadings are related to leaf traits.

Table S1: Key to event ID’s and short names

Event names are the initial transformation event identities from the initial large-scale field trial. Short names are abbreviated as follows. C = non-transgenic control, TS = transgenic sterile (RNAi-LFY trees with sterile catkins), TN = transgenic normal (RNAi-LFY trees with normal catkins).

Event ID	Short name
control	C
17	TS1
139-1	TS2
143	TN1
152	TN2
194	TN3
126-3	TN4

Table S2: Summary of p-values from likelihood ratio and Chi-squared analysis of flowering and leaf flush traits Comparisons between groups are shown with a – sign, such as TS-TN. Significant values (<.05) are shown in bold.

Likelihood Ratio Test	**Early Flowering Score**	**Late Flowering Score**	**Flowering (Y/N)**	**Leaf Flush (top)**	**Leaf Flush (bottom)**	**Max Flowering Score**	**Sum of Flowering Scores**
Event	**<.0001**	**<.0001**		**0.0094**	0.38	**<.0001**	**<.0001**
Block	0.35	0.24		**0.0012**	**0.034**	0.18	0.66
Chi-squared							
TS-TN-C	**<.0001**	**<.0001**	0.07	**0.0098**	0.50	**0.00018**	**<.0001**
TS-TN	**<.0001**	**<.0001**	0.17	0.11	0.50	**0.00058**	**<.0001**

Table S3: Percent change between event means for block-adjusted 2019 field data
Percent change was calculated as the event mean minus the trait mean divided by the trait mean times 100. Negative signs indicate that the event mean was smaller than the trait mean.

Percent change between event means	Early Flowering	DBH (cm)	Height (cm)	Volume cm^3	Leaf Flush (top)	Leaf Flush (bottom)	Max Flowering Intensity	Late Flowering
C	41.25	-12.06	-11.47	-8.93	14.43	10.87	-15.91	-16.89
TN1	56.67	22.62	12.00	30.62	19.69	18.31	14.86	22.41
TN2	49.19	5.33	5.12	10.96	-1.06	-5.86	13.17	17.63
TN3	47.33	-1.66	-1.24	-6.04	-4.29	-9.23	37.08	33.73
TS1	-100.63	-13.89	-6.87	-21.37	-21.98	-14.96	-1.37	-5.82
TS2	-99.06	-7.08	1.29	-14.15	-8.48	-0.63	-47.90	-52.24

Percent change between event means	Sum of Floral Scores	Flowering (Y/N)	Leaf Area	Petiole Length	Petiole Width	Petiole L/W Raito	Leaf Weight	Leaf-specific Weight
C	-9.47	-7.52	-2.22	-5.00	-8.38	1.76	-5.17	-3.63
TN1	50.28	7.67	11.65	6.43	-2.98	11.92	10.06	-1.19
TN2	28.78	2.77	14.80	11.58	3.01	7.64	22.23	6.77
TN3	48.80	2.10	0.17	3.88	4.11	-1.55	1.51	0.62
TS1	-43.30	2.35	-13.11	-9.62	0.68	-9.79	-19.87	-7.00
TS2	-77.92	-8.06	-13.16	-8.14	4.15	-11.61	-10.67	4.43

Table S4: Numbers of trees in each block, event, and group at time of planting in 2016

	TS1	TS2	TN1	TN2	TN3	TN4	C	Total
**Border trees**	13	15	13	12	10	1	5	**69**
**Block 1**	5	5	5	5	5	5	5	**35**
**Block 2**	6	6	6	6	5	0	6	**35**
**Block 3**	6	6	6	6	5	0	6	**35**
**Block 4**	5	6	5	5	5	3	6	**35**
**Showcase**	2	3	2	2	2	0	2	**12**
**Event Total**	37	40	37	36	32	9	30	
**Group Total**	**77**		**114**				**30**	**221**
